# Immunometabolism: Molecular Mechanisms, Diseases, and Therapies 2018

**DOI:** 10.1155/2019/2340914

**Published:** 2019-01-30

**Authors:** José C. Rosa, Fabio S. Lira, William Festuccia, Barbara Wessner, Nicolette C. Bishop

**Affiliations:** ^1^Department of Cell Biology and Development, Institute of Biomedical Sciences, University of Sao Paulo, Sao Paulo 05508000, Brazil; ^2^Exercise and Immunometabolism Research Group, Department of Physical Education, Universidade Estadual Paulista (UNESP), Presidente Prudente, SP 19060-900, Brazil; ^3^Department of Physiology and Biophysics, Institute of Biomedical Sciences, University of Sao Paulo, Sao Paulo 05508000, Brazil; ^4^Centre for Sport Science and University Sports, Vienna, Austria; ^5^School of Sports, Exercise and Health Science, Loughborough University, Loughborough, UK

This third edition of this special issue focused on the interrelationships between metabolic pathways, metabolites, and the immune system. Chronic low-grade inflammation is a common phenotype found in several diseases including obesity, cancer, type 2 diabetes, and cardiovascular diseases, directly participating in their development, as well as a connecting factor between them. Evidence accumulated over the years has shed light on the important role of dysregulated metabolic pathways caused by nutrient excess, lipid overload, sedentary behaviour, and aging as promoting factors and modulators of inflammation. Immune cells through the secretion of cytokines and other inflammatory mediators, on the other hand, also modulate insulin signalling, glucose, and lipid metabolism.

This special issue received the submission of 20 manuscripts focused on different aspects of the intricate relationship between the immune system and metabolism; among which, 9 were accepted for publication. Three of these articles provide insightful reviews of the literature within the scope of this issue. Yamashita and colleagues, for example, reviewed the molecular mechanisms by which chronic exposure to excessive amounts of the nutrient lipids, glucose, and amino acids regulates inflammatory pathways, with a special emphasis in the role of the nutrient and energy sensors mTOR, AMPK, and PPARs in this context. Belizário and colleagues, on the other hand, reviewed the emerging role of microbiota as a major factor in the development of chronic inflammatory diseases. This timely review article, among other aspects, discussed the role of dysbiosis as the trigger of inflammation in different pathological conditions and the possibility of management of microbiota as a nonpharmacological intervention to counteract disease development. Finally, Brinchmann and colleagues elegantly revisited the role of galectins, *β*-galcotosid-binding lectin found in the intra- and extracellular compartments, in the regulation of metabolism and inflammation and as possible targets to treat chronic diseases.

In addition to review articles, this special issue also published 6 interesting original studies. Among them, 4 described possible beneficial effects of anti-inflammatory strategies to counteract disease development. Lin et al., for example, demonstrate that shock wave therapy induces mitochondrial delivery to lung parenchyma and, by reducing alveolar macrophage infiltration and fibrosis, protects from acute respiratory distress syndrome. In another original study, Qian et al. robustly showed that osthole, a natural coumarin extract, reduces inflammation and the production of proinflammatory cytokines by macrophages increasing mice survival to septic shock. In the same direction, Samblas and colleagues elegantly demonstrate that folic acid, a naturally occurring dietary component of the methionine pathway for the synthesis of S-adenosyl methionine (SAM), the universal methyl-donor for DNA methylation, reduces the production and secretion of the proinflammatory cytokines TNF-*α* and IL-1*β* induced by LPS in macrophages. Finally, Wang et al. showed that inhibition of the histone deacetylase HDAC2 with CAY10683 protected rats from LPS-induced acute liver failure and endotoxemia by improving the integrity of the intestinal barrier and reducing the activation of the LPS-TLR4-MYD-88 pathway.

Noteworthy, two original studies of this special issue have evaluated the changes in the metabolic profile of immune cells in different conditions. Ahmed and colleagues demonstrate through transcriptional profile dataset that treatment of human and mice macrophages with interferon- (IFN-) *α* promotes important changes in their metabolic signature characterized by activation of pathways involved in cellular bioenergetics, cellular oxidant status, cAMP/AMP and cGMP/GMP ratios, branched chain amino acid catabolism, cell membrane composition, fatty acid synthesis, and *β*-oxidation. Finally, Santarsiero and colleagues elegantly showed that patients with Behçet's syndrome (BS), a multisystemic disorder characterized by chronic inflammation and vasculitis, displayed elevated mRNA levels of the mitochondrial citrate carrier (SLC25A1) and ATP-citrate lyase (ACLY) in peripheral blood mononuclear cells (PBMCs) suggesting a dysregulation of citrate metabolism that could participate in the increased proinflammatory response displayed by these cells.

Altogether, the studies published in this special issue bring new insights into the intricate mechanisms driving the inflammatory processes associated with metabolic diseases. We hope that these studies will pave the way for the development of novel efficient strategies to prevent and treat these increasingly common conditions.

